# SACROILIAC JOINT SYNOSTOSIS AS AN ATYPICAL CAUSE OF SPORTS-RELATED GROIN PAIN: A CASE REPORT

**DOI:** 10.2340/jrm.v58.45687

**Published:** 2026-04-21

**Authors:** Lina VAN BRABANDER, Tom NONNEMAN, Marc SCHILTZ, Bernard FLORÉ, Samar M. HATEM

**Affiliations:** 1 Department of Physical Medicine and Rehabilitation, Universitair Ziekenhuis Brussel, Brussels; 2 Department of Physical Medicine and Rehabilitation, AZ Sint-Maarten, Mechelen; 3 Department of Radiology, AZ Glorieux, Ronse; 4 Faculty of Medicine and Pharmacy, Vrije Universiteit Brussel, Brussels; 5 Stimulus Research Group, Vrije Universiteit Brussel, Brussels, Belgium

We present the case of a 50‑year‑old male recreational triathlete with an uncommon aetiology of groin pain, due to iliopsoas muscle friction over an anterior synostosis of the sacroiliac joint. MRI images are remarkable in demonstrating the underlying pathology.

To date, we have found no previously reported cases of this condition in the literature. We found 1 case of impingement of the psoas muscle by a lumbar disc osteophyte (1).

## CASE REPORT

Our patient is a 50-year-old male recreational triathlete without relevant medical history, performing training runs of between 10 and 20 km, almost daily.

This patient first presented to the surgical department with right-sided groin pain. A reducible swelling in the right groin was noticed. Ultrasound confirmed an indirect inguinal hernia, for which a Lichtenstein procedure was performed. Postoperative follow-up was unfavourable, characterized by persistent pain in the right groin radiating towards the right iliac fossa. A follow-up ultrasound demonstrated soft tissue swelling around the incision site without evidence of hernia recurrence.

A diagnostic test with daily pregabalin 75 mg was carried out for at least 3 weeks, because the distinction between types of postoperative pain was not straightforward. Persistent localized postoperative pain may be nociceptive or neuropathic in nature and may be caused by local inflammation, nerve lesions, or due to irritation by the mesh (2–5). In our patient, pregabalin did not improve symptoms, thus suggesting that the pain was not neuropathic.

Due to continuing complaints, the patient was then assessed at the department of Physical Medicine and Rehabilitation (PMR). He reported persistent movement-related right-sided groin pain, predominantly occurring after prolonged runs. The pain occurred after each single run, progressively increasing until the patient was able to rest.

*Clinical examination.* Physical examination revealed limited internal rotation of both hips, bilaterally shortened quadriceps and iliopsoas muscles, and pain when contracting the right-sided external oblique muscle. The incision scar was tender upon palpation. Examination of the lumbar spine and neurological examination of the lower limbs were normal.

*Imaging and initial management*. The first pelvic MRI demonstrated mild inflammatory changes around the scar. A pelvic X-ray revealed subtle signs of femoroacetabular impingement (FAI) of the CAM type, accompanied by mild degenerative changes of the femoroacetabular joint.

A course of oral non-steroidal anti-inflammatory drugs (NSAID) resulted in an improvement of scar-related pain. However, the right-sided groin pain persisted after prolonged running. Physiotherapy targeting FAI was initiated, which focused on mobilizing both hips, strengthening the gluteal musculature, and improving core stability.

*Clinical course*. Over the course of 6 months, symptoms worsened and occurred during runs. The patient was unable to maintain his usual training scheme. Secondary complaints developed, including lumbar pain and bilateral trochanteric discomfort. Clinical assessment showed a persistent limitation of hip internal rotation and bilateral shortened quadriceps and iliopsoas muscles. Now, right-sided groin pain was elicited during end-of-range hip flexion, accompanied by a positive C-sign. The C-sign is a clinical gesture in which the patient localizes hip pain by forming a “C” shape with the thumb and index finger while grasping the lateral aspect of the hip. This manoeuvre is frequently associated with deep internal joint pain of the hip (6). The physiotherapist related an unfavourable progression of symptoms. Pain occurred predominantly when the right leg acted as the swing limb rather than as the stance limb, which is atypical for intra-articular pain. Symptoms were aggravated by active and passive hip flexion–extension.

**Fig. 1:**
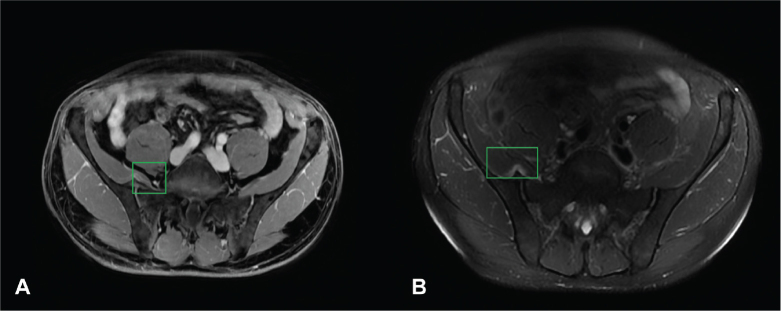
(A) MRI axial Lava-Flex Dixon Water – Gd. (B) MRI axial T2-weighted fat saturated. The imaging demonstrates a synostosis located on the anterior aspect of the sacroiliac joint at the right side, impinging upon the iliopsoas muscle. Associated oedema within the muscle belly is clearly evident as a white area around the synostosis (box: synostosis and oedema).

**Fig. 2:**
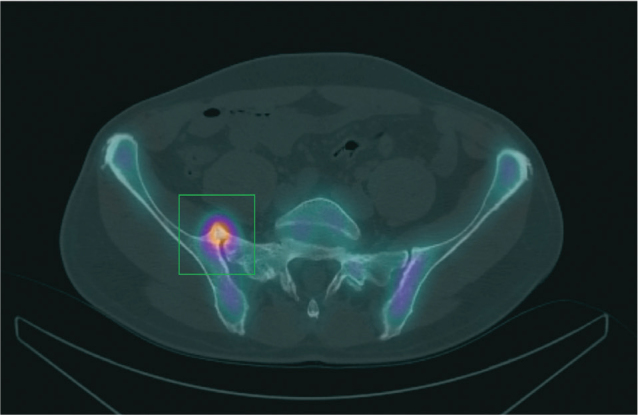
SPECT – CT. This axial SPECT/CT image (fused image of scintigraphy with computed tomography) demonstrates a marked tracer uptake/accumulation at the synostosis at the right sacro-iliac joint (box: tracer accumulation at the synostosis).

After consultation with the radiologist, a second pelvic MRI was performed with a larger field of view. This MRI revealed a marked synostosis at the anterior sacroiliac joint (SIJ), accompanied by significant soft tissue oedema involving the iliopsoas muscle (Fig. 1). A complementary bone scan demonstrated intense uptake at the site of the anterior synostosis of the right sacroiliac joint (Fig. 2).

The patient then consulted an orthopaedic surgeon, who proposed an intra-articular hip infiltration with a corticosteroid. This intervention did not result in clinical improvement.

At this stage the clinical history, symptoms and signs, and diagnostic work-up suggest a diagnosis of repetitive friction of the iliopsoas muscle over the anterior surface of the sacroiliac joint during hip flexion–extension movements while running.

The exercise programme was subsequently adapted. Movements provoking hip flexion–extension were minimized, and alternative activities were introduced to maintain cardiovascular fitness. The aim was to reduce friction between the synostosis and the iliopsoas muscle and to allow resolution of the iliopsoas’ inflammatory response. Running was resumed upon the patient’s insistence, taking care to limit volumes and avoid provoking pain.

Due to the synostosis being permanent, the prognosis is considered poor. Surgery is not recommended in this location.

### Diagnostic assessment and challenges

**Fig. 3:**
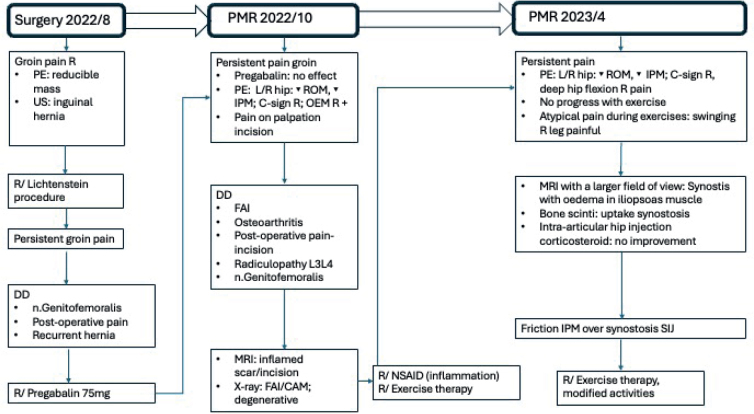
Timeline diagnostic assessment. PE: physical examination; US: ultrasound; DD: differential diagnosis; R: right; L: left; OEM: musculus obliquus externus abdominis; R/: therapy; ROM: range of motion; IPM: musculus iliopsoas.

Fig. 3 illustrates the timeline of the diagnostic work-up.

Initially, persistent postoperative pain or injury to the genital branch of the genitofemoral nerve was considered (4, 5). The first postoperative ultrasound excluded a recurrence of the inguinal hernia, and the initial pelvic MRI showed only soft tissue inflammation at the incision site. Additional pelvic radiographs, prompted by limited hip internal rotation, revealed bilateral CAM-type lesions with early degenerative changes of the femoro-acetabular joints. The working diagnosis at this stage was FAI/CAM morphology with mild bilateral hip osteoarthritis as the cause of symptoms. Further imaging was requested due to persistent complaints despite physiotherapy and to atypical findings during rehabilitation sessions. After consultation with the radiologist, a second pelvic MRI and bone scintigraphy were performed. Broader imaging revealed a large anterior synostosis at the right sacroiliac joint with overlying oedema in the right iliopsoas muscle. SPECT-CT demonstrated marked tracer uptake at this site.

The diagnostic process presented several challenges. As the patient had previously undergone inguinal hernia repair, postoperative pain was initially assumed to be the primary cause (4, 5). Clinically, pain was localized along the incision scar, with additional signs suggestive of osteoarthritis or FAI (2, 3). MRI and radiography supported this hypothesis.

Only after treatment failure of targeted physiotherapy, physiotherapist feedback, and radiological consultation was broader imaging performed. This revealed a rare cause of groin pain: a large anterior synostosis across the SIJ, associated with oedematous iliopsoas muscle.

## DISCUSSION

Strengths of this case include the striking images on MRI and the significant uptake on bone scintigraphy. The absence of any benefit from intra-articular hip corticosteroid injection further supported the hypothesis of iliopsoas friction over the sacroiliac synostosis.

In retrospect, the symptoms were partly compatible with FAI (C-sign, groin pain, activity-related pain), but the gait pattern – pain occurring predominantly when the right leg acted as the swing limb rather than the stance limb – was incongruent (2, 3).

A limitation of this case report is that the patient was not assessed in the Department of Physical and Rehabilitation Medicine prior to the inguinal hernia repair. It is therefore difficult to determine whether the initial complaints were identical to later symptoms, or if the hernia surgery played a role in the subsequent clinical course (4). It also remains uncertain whether the inguinal hernia caused the original inguinal pain or was merely an incidental finding.

The most frequent causes of groin pain in athletes include osteoarthritis, femoroacetabular impingement, sports hernia, abdominal muscle injuries, stress injuries of the pubic bone or femoral neck, adductor muscle injuries, nerve entrapments, and, specifically after inguinal surgery, injury to the genital branch of the genitofemoral nerve or L1–L3 radiculopathies (2, 3).

The key lesson from this case is the importance of re-evaluating persistent symptoms, maintaining close interaction with physiotherapists, and actively involving radiologists in complex cases.
